# Food Adulteration

**Published:** 1897-08

**Authors:** 


**Food Adulteration.**—The *Lancet-Clinic* says: "The time has come when some additional laws should be enacted, whereby the contents of every package of food product offered for sale to the public should be stamped with the name of its contents, and every package of medicine sold on other than a registered physician's prescription should be marked distinctly in legible print with the formula of its contents. Such a law would save to the people every year hundreds of thousands of dollars. Such a law would have quite a perceptible influence over the sale of fraudulent medicines, including consumption cures, which are held out as working through the aid of divine Providence, and thereby warranted to not only cure the incurable, but which are addressed as a solace to those who have entered the bourne from whence no traveller ever returns."

---

**An interesting** article on "Caracature a la Salle de Garde" appears in a late number of *Le Correspondent Medical.* One poem on the microscope is enough to warrant a poor English rendering:

'Tis well the infinitely small
Has not the one same charm for all.
To every mind doth not appear
An ocean in a single tear.
Who then will dread gaunt forms Cyclopian?
Only a mind that's microscopian.
Now as for us, it seems not wise
To gaze through pathological eyes.
To see germ chickens in each coop,
See hairs in every dish of soup;
No matter, oyster, beef and clam,
We'll swallow all and say "Be damn!"

—Correspondent in *Lancet-Clinic.*

